# Trend of HIV transmitted drug resistance before and after implementation of HAART regimen restriction in the treatment of HIV-1 infected patients in southern Taiwan

**DOI:** 10.1186/s12879-019-4389-1

**Published:** 2019-08-23

**Authors:** Ya-Wei Weng, I-Tzu Chen, Hung-Chin Tsai, Kuan-Sheng Wu, Yu-Ting Tseng, Cheng-Len Sy, Jui-Kuang Chen, Susan Shin-Jung Lee, Yao-Shen Chen

**Affiliations:** 1 Division of Infectious Diseases, Department of Medicine, Kaohsiung Veterans General Hospital, Kaohsiung, Taiwan; 2 Faculty of Medicine, School of Medicine, National Yang-Ming University, Taipei, Taiwan; 3 Department of Parasitology, Kaohsiung Medical University, Kaohsiung, Taiwan; 4 Institute of Biomedical Sciences, National Sun Yat-Sen University, Kaohsiung, Taiwan

**Keywords:** HIV, Drug resistance, Treatment naïve

## Abstract

**Background:**

The use of fixed combination antiretroviral therapy with a low genetic barrier for the treatment of patients infected with human immunodeficiency virus (HIV) may affect the local HIV transmitted drug resistance (TDR) pattern. The present study aimed to investigate changes in the prevalence of HIV TDR following the implementation of a fixed regimen of HIV treatment in Taiwan in 2012.

**Methods:**

TDR was measured in antiretroviral treatment-naïve HIV-1-infected individuals who participated in voluntary counseling and testing between 2007 and 2015 in southern Taiwan. Antiretroviral resistance mutations were interpreted using the HIVdb program from the Stanford University HIV Drug Resistance Database.

**Results:**

Sequences were obtained from 377 consecutive individuals between 2007 and 2015. The overall prevalence rates of TDR HIV among the study population from 2007 to 2011 and 2012–2015 were 10.6 and 7.9%, respectively. Among the detected mutations, K103 N and V179D + K103R were more frequently observed after 2012. Four HIV-infected patients with K103 N variants were detected after 2012, and 4 of the 5 patients with V179D + K103R variants were found after 2012. No significant differences were observed in the TDRs among nucleoside reverse transcriptase inhibitors (NRTIs), non-NRTIs (NNRTIs), protease inhibitors, multiple drug resistance, and any drug resistance between period 1 (2007–2011) and period 2 (2012–2015).

**Conclusions:**

A fixed treatment regimen with zidovudine/lamivudine + efavirenz or nevirapine as first-line therapy for treatment-naïve patients infected with HIV did not significantly increase the TDR during the 4-year follow-up period. Due to the increase in NNRTI resistance associated with mutations after 2012, a longer follow-up period and larger sample size are needed in future studies.

## Background

Antiretroviral drugs are now widely available for individuals living with human immunodeficiency virus (HIV) worldwide. However, the emergence of transmitted HIV drug resistance can substantially increase a patient’s chance of treatment failure [1–3].

Estimated rates of transmitted drug resistance (TDR) in HIV vary globally, possibly due to differences in risk exposure categories, the duration of antiretroviral therapy (ART) available in the study population, and the time from seroconversion [4–6]. The extent of drug resistance increases with the length of treatment [5, 7], and TDR is driven by both patients who are naïve to and fail ART [8]. A previous study showed that first-line therapy with efavirenz (EFV) plus zidovudine (AZT) paired with lamivudine (3TC) or emtricitabine (FTC) was associated with an increased incidence of drug resistance [9], while initial therapy with boosted protease inhibitor (bPI)-based regimens has been reported to result in less resistance within and across drug classes [10]. Therefore, treatment of HIV-infected patients with a fixed therapy regimen with a low genetic barrier may affect the local HIV TDR pattern.

In Taiwan, HIV infection is a reportable disease. Since the first HIV-1 infected patient was diagnosed in Taiwan in 1984, the annual number of reported cases has increased every year. A total of 31,036 adults were reported as being infected with HIV-1, by the end of 2015, most of whom (18,079, 58.25%) were men who have sex with men (MSM) or were bisexual. This group continues to have a disproportionately high burden of HIV infection both in Taiwan and globally [11]. The Taiwan Center for Disease Control (CDC) has provided voluntary counseling and testing (VCT) services since 1997 to reach the target populations most at risk of HIV infection, and the positive rate of HIV from VCT services is around 2.1~4.7% in Taiwan. In addition, ART has been provided free of charge since April 1997 after reporting HIV infection to the government. However, routine drug resistance testing has not been available to clinicians. According to a previous epidemiological study, the TDR rate is 8.0–11.1% in Taiwan [12–14]. HIV-infected Taiwanese patients receive free HIV care based on the national treatment guidelines. Before June 2012, clinicians could choose antiretroviral drugs for HIV-infected patients according to their clinical judgement, and all available antiretroviral drugs could be prescribed by clinicians. Nucleoside reverse transcriptase inhibitors (NRTIs), non-nucleoside reverse transcriptase inhibitors (NNRTIs, including EFV and nevirapine), protease inhibitors (PIs, including atazanavir, saquinavir, nelfinavir, lopinavir, tipranavir, ritonavir and darunavir), integrase strand transfer inhibitors (INSTIs, including raltegravir) and maraviroc are all available in Taiwan. From June 2012 to June 2016, a fixed regimen with AZT/3TC plus EFV or nevirapine (NVP) was suggested by the Taiwan national treatment guidelines. Clinicians must follow these guidelines, and the third agent must be EFV or NVP. Therefore, if HIV-infected patients were not hepatitis B virus (HBV) carriers, they would receive AZT/3TC plus EFV or NVP as the first-line therapy during this time period. For HIV and HBV coinfected patients, tenofovir (TDF)/3TC or TDF/FTC plus EFV or NVP was required as first-line therapy. The present study aimed to investigate the prevalence of transmitted HIV drug resistance before and after the implementation of the fixed regimen with a low genetic barrier for HIV treatment.

## Methods

### Ethics statement

All participants were informed of the study procedures and provided written informed consent prior to their inclusion. The present study was approved by the Institutional Review Board of Kaohsiung Veterans General Hospital (KVGH; approval Nos. VGHKS97-CT3–14, VGHKS98-CT1–08 and VGHKS15-CT5–10).

### Study population

KVGH has provided VCT services since 1997. The positive rate of HIV over the past 10 years has been 2.4–5.4%, and most cases have been MSM or bisexual. HIV sequencing and genotypic resistance analysis were performed if the patients returned to the hospital to receive further disease surveillance and management. The CD4 cell count and plasma viral load were checked when the patient returned to the clinic after a positive HIV infection was confirmed. Free testing was also offered for HBV and hepatitis C virus (HCV). In total, 415 patients who tested positive on HIV ELISA or rapid test came back to our hospital for further care and were then enrolled into this study. Thirty-eight patients did not have sequencing results due to insufficient plasma, low viral load or interfering substances in the blood. HIV sequencing and genotypic resistance data were available for 377 patients. These 377 patients with newly diagnosed HIV infections were then enrolled into the study for further analysis between 2007 and 2015.

### Patient and public involvement

The participants were not involved in the design or conduct of this study. In addition, no patient advisers were involved in this study. Since the fixed regimen policy with AZT/3TC plus EFV or NVP already changed, there was no plan to disseminate the results to the study participants.

### Serological tests for hepatotropic viruses and CD4, and the measurement of viral load

HBV surface antigen (HBsAg) and anti-HCV antibodies were detected using an HBsAg radioimmunoassay (ARCHITECT i1000SR; Abbott, Abbott Park, IL, USA) and anti-HCV ELISA kit, respectively. The plasma HIV RNA load and CD4 cell count were quantified using a Cobas Amplicor HIV-1 monitor test, version 1.5 (Roche Diagnostics, Indianapolis, IN, USA) and FACSFlow (BD Biosciences, Franklin Lakes, NJ, USA), respectively.

### *Reverse transcription-polymerase chain reaction (RT-PCR*) amplification and resistance testing

Protease and reverse transcriptase were sequenced on the basis of HIV-amplification products using Viroseq version 2.8 (Celera; Quest Diagnostics, Secaucus, NJ, USA) [15]. For integrase sequencing, the integrase region spanning codons 1–288 was targeted, using the following nested-RT-PCR primers: Int1 forward, 5′- CAT GGG TAC CAG CAC ACA CAA AGG − 3′ and Int1 reverse, 5′- CCA TGT TCT AAT CCT CAT CCT GTC − 3′ for the first PCR round, and Int2 forward 5′- GGA ATT GGA GGA AAT GAA CAA GTA GAT − 3′ and Int2 reverse 5′- GCC ACA CAA TCA TCA CCT GCC ATC − 3′ for the second round [16].

To make comparisons with other studies, the antiretroviral resistance mutations were interpreted using the HIVdb program from the Stanford University HIV Drug Resistance Database (https://hivdb.stanford.edu/, Version 7.0) [17]. Patients classified as having low-level, intermediate and high-level resistance were defined as having drug resistance. Multidrug resistance was defined as having genotypic resistance to more than one class of anti-retroviral agent. Each step was performed with negative controls.

### Statistical analysis

Statistical analysis was performed using SPSS version 21.0(IBM Corp., Armonk, NY, USA). Descriptive statistics included frequency analysis (percentages) for categorical variables and medians with the interquartile range (IQR) for continuous variables. Categorical variables were compared using Pearson’s or Fisher’s chi-square test, and non-categorical variables were compared using the independent samples t-test. Continuous data were analyzed using linear regression. All tests were two-tailed, and a *P-*value of < 0.05 was considered to indicate a statistically significant difference.

## Results

### Patient characteristics

Between 2007 and 2011(period 1), 161 patients were positively diagnosed with HIV infection through VCT at KVGH. The median age of the 161 HIV treatment-naive patients was 26 years (IQR, 22–32 years). The median CD4 count was 347/cumm (IQR, 230–445/cumm) and the median viral load was (log_10_) 4.7 copies/mm^3^ (IQR, 4.2–5.0 copies/mm^3^). Analysis of the nucleotide sequences from the protease and reverse transcriptase regions showed that 98.1% of the cases were infected with HIV subtype B. A total of 216 patients were positively diagnosed with HIV infection between 2012 and 2015 (period 2). Men who have sex with men accounted for 92% of the patient group. The median CD4 count was 295/cumm (IQR, 200–436/cumm), and the median viral load was (log_10_) 4.8 copies/mm3 (IQR, 4.4–5.2 copies/mm^3^). A comparison of the baseline characteristics between the patients diagnosed before and after 2012 is presented in Table 1.

**Table 1:** Baseline characteristics before and after the fixed ART regimen was implemented in 2012

Baseline demographic data	Time period		*P*-value
	2007–2011 (*N* = 161)	2012–2015 (*N* = 216)	
Median age (IQR), years	26.0 (22.0–32.0)	26.0 (23.0–31.0)	0.73
Male (%)	160 (99.3)	215 (99.5)	0.83
Median CD4 count (IQR), cells/mm^3^	347 (230–445)	295 (200–436)	0.25
Median viral load (IQR), Log_10_ copies/mm^3^	4.7 (4.2–5.0)	4.8 (4.4–5.2)	0.02
HIV subtype B (%), *n*	158 (98.1)	209 (96.8)	0.41
Co-infection			
HBV carrier (%), *n*	16 (10.1)	20 (9.3)	0.78
Anti-HCV Ab(+) (%), *n*	3 (1.9)	6 (2.8)	0.59
Sexual orientation, %			
MSM	86.9	92.0	0.08
Heterosexual	13.1	8.0	

*ART* anti-retroviral therapy, *HIV* human immunodeficiency virus, *HBV* hepatitis B virus, *HCV* hepatitis C virus, *IQR* interquartile range, *MSM* men who have sex with men

### Prevalence of TDR

**Fig. 1:**
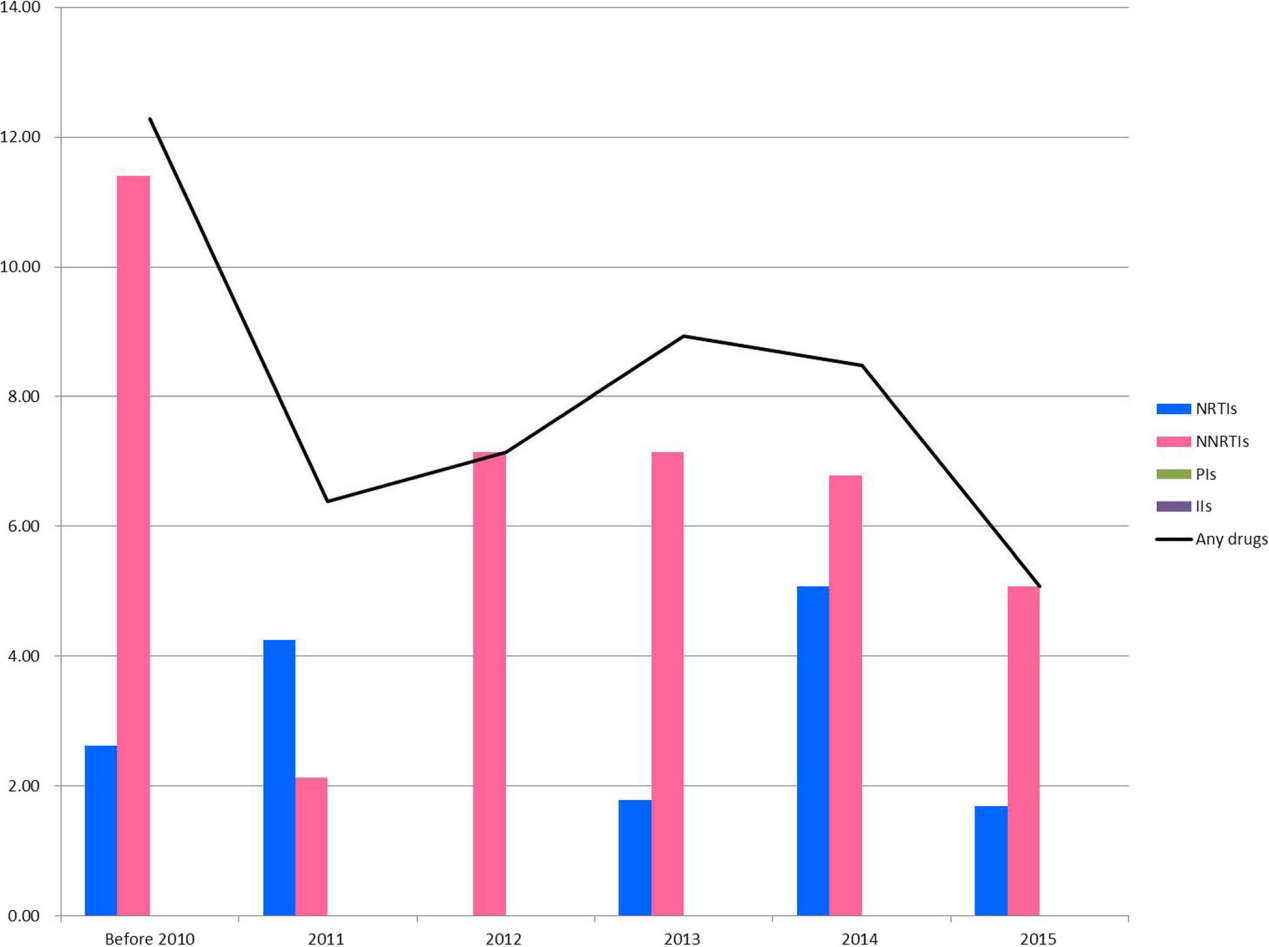
Frequency of resistance to NRTIs, NNRTIs, PIs and INSTIs between 2007 and 2015(data for INSTIs were only available from 2013). NRTI, nucleoside reverse transcriptase inhibitor; NNRTI, non-nucleoside reverse transcriptase inhibitor; PI, protease inhibitor; INSTI, integrase strand transfer inhibitor

**Fig. 2:**
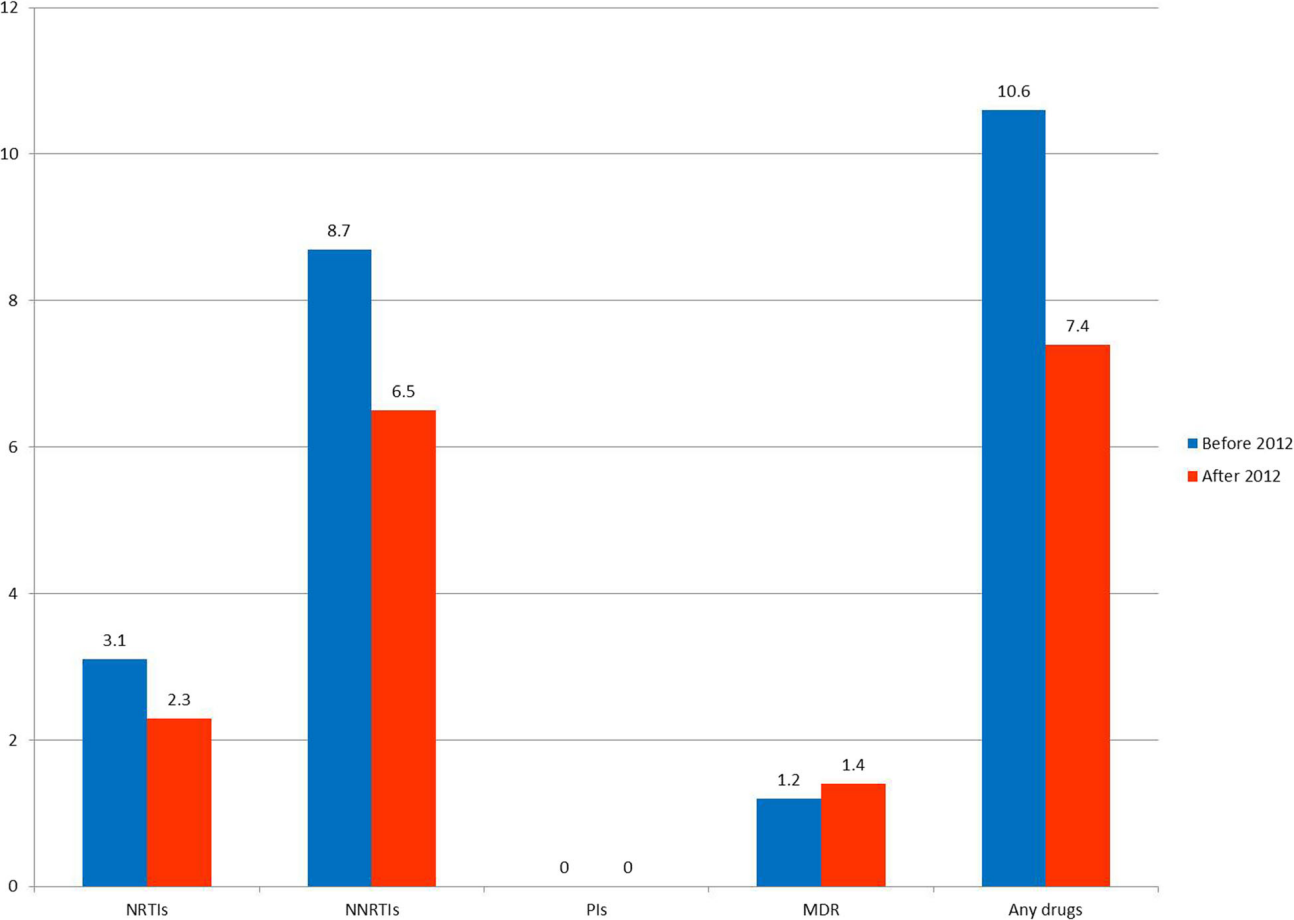
Comparison of transmitted drug resistance between fixed and flexible regimens for HIV management (flexible regimen, before 2012; fixed regimen, after 2012). The *P*-values were 0.75, 0.43, > 0.99 and 0.36 for NRTIs, NNRTIs, multi drug resistance and any drugs, respectively. NRTI, nucleoside reverse transcriptase inhibitor; NNRTI, non-nucleoside reverse transcriptase inhibitor

The overall prevalence rates of TDR HIV in the study populations diagnosed in period 1 and 2 were 10.6 and 7.9%, respectively. In both periods, the majority of the detected drug resistance mutations conferred resistance to a single class of antiretroviral drugs, which was most commonly NNRTIs. The frequency of resistance to NRTIs, NNRTIs, PIs and INSTIs in the patients between 2007 and 2015 is shown in Fig. 1. Integrase resistance mutations were surveyed after 2013, and 174 patients were enrolled for analysis. However, in the present study, no patients harbored integrase resistance-associated mutations. The annual prevalence of TDR HIV was stable between 2009 and 2015 (slope = − 0.086; data from 2007 and 2008 were excluded due to extreme values and small samples sizes). A comparison of TDR in the patients either treated with a fixed or flexible regimen for HIV is shown in Fig. 2. Although the TDR seemed to be lower after the fixed regimen was introduced (2012–2015), there was no significant difference in TDR for NRTIs, NNRTIs, PIs, multiple drug resistance, and any drug resistance between the two time periods.

Most of the collected samples harbored TDR-associated mutations for NRTIs and NNRTIs. The mutations which contributed to drug resistance against NRTIs and NNRTIs were quite diverse. The NRTI mutations included K65R (0.27%), D67N (0.27%), L74 V (0.27%), M184 V (1.06%), L210 W (0.2%) and T215S (0.53%). For NNRTIs, the most prevalent drug resistance mutations were K103 N (1.59%), V179D + K103R (1.33%), Y181C (0.80%), V108I (0.53%), Y188L (0.53%), G190A (0.53%), H221Y (0.53%), Y318F (0.53%), A98G (0.27%), V106A (0.27%), E138A (0.27%), E138R (0.27%), Y188C (0.27%) and M230 L (0.27%).

**Fig. 3:**
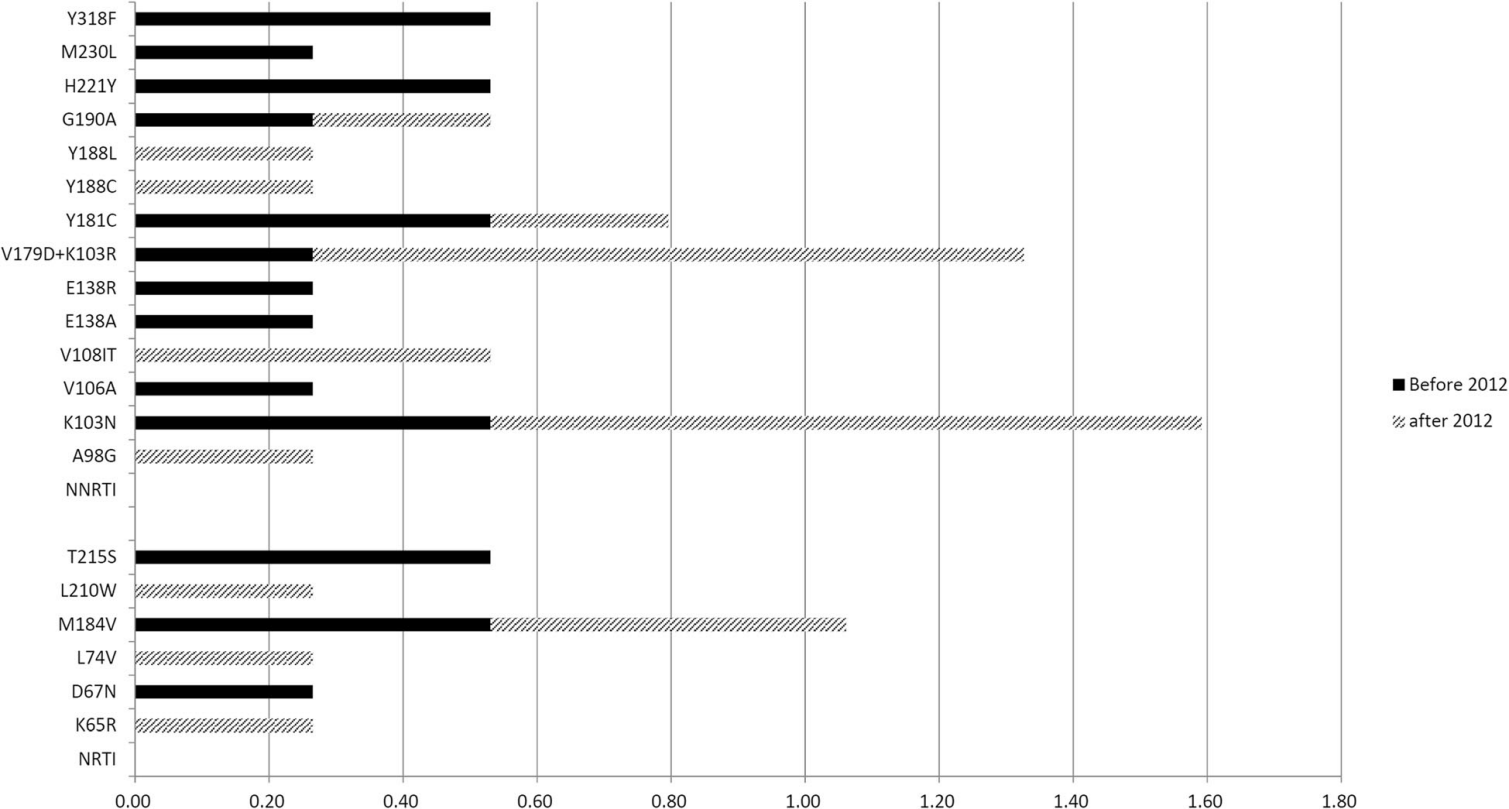
Percentage of patients with specific mutations, by drug class

Among these detected mutations, K103 N and V179D + K103R were observed more frequently after 2012. Two-thirds of the HIV-infected patients who harbored K103 N variants were detected after 2012, and four-fifths of the patients with V179D + K103R variants were detected after 2012. The percentage of patients with specific mutations is shown in Fig. 3.

## Discussion

In this 9-year surveillance study of TDR in HIV-1 strains, we found that the prevalence of antiretroviral resistance mutations was stable in southern Taiwan, including after the introduction of a fixed regimen with AZT/3TC plus EFV or NVP as first-line therapy. Between June 2012 and December 2015, ART comprised of dual NRTIs (AZT/3TC) combined with an NNRTI (EFV or NVP) as a third agent was prescribed as the first-line therapy for HIV-infected patients according to the national treatment guidelines in Taiwan. This was despite the fact that a previous review had proven that HIV-infected patients who received these regimens had higher rates of resistance to NRTIs and NNRTIs compared to those given bPIs as a third agent [10]. The present study focused on TDR in HIV, and found that the HIV TDR rate has remained stable following the introduction of a fixed regimen, indicating that it was not affected by the policy. However, significant differences in HIV TDR may not have been seen due to the relatively short follow-up period after the introduction of the fixed regimen. Furthermore, the time to initiation of ART also changed during the study period. The initiation of ART in patients with a CD4 count < 200 cells/cumm was suggested in 2006, however the initiation of ART was recommended in patients with a CD4 count < 350 cells/cumm in 2010, and in 2013 the suggested cut-off value was amended to < 500 cells/cumm. Several studies have demonstrated that early ART can prevent HIV-1 transmission in HIV-discordant couples [18–20]. In addition, several studies have supported that case management services improve health outcomes. [21, 22] In 2008, the Taiwan CDC initiated an HIV case management program in AIDS-designated hospitals to provide integrated services and risk reduction counseling for HIV-infected individuals. Between 2007 and 2015, the number of AIDS-designated hospitals increased, and the application of case management programs become widespread. These improvements could offer better care quality for HIV-infected patients. Therefore, the relatively stable trend of TDR in Taiwan, even under a fixed regimen, may be due to the early administration of ART and comprehensive case management programs.

In line with several cross-sectional studies conducted in the USA and Europe [23–25], the present study did not identify any major INSTI mutations among the antiretroviral-naïve HIV-1 positive patients from southern Taiwan. Major INSTI mutations have been previously detected in treatment-naïve HIV-infected patients from Taiwan [8], however only one strain has been found to harbor Q148R. Another study in northern Taiwan showed that the prevalence of INSTI-resistant HIV-sequences was 0.6% among ART-naïve patients [26]. In Taiwan, raltegravir has been available for clinical use in treatment-naïve patients since 2012. In addition, dolutegravir has only been available for treatment-naïve patients since December 2015. The low resistance rate to INSTIs among the study populations may be due to a reduced exposure to INSTIs in HIV-infected patients in Taiwan. As dolutegravir is recommended as first-line therapy in several guidelines, including the Taiwan HIV treatment guidelines (since June 2016), the prevalence of transmitted INSTI mutations may have increased over time. Therefore, regular public health surveillance to actively monitor INSTI resistance may be necessary.

In addition, no resistance to PIs was identified in the patients included in the present study. Several PIs have been available in Taiwan since 2008, including atazanavir, saquinavir, nelfinavir, lopinavir, tipranavir, ritonavir and darunavir, and the lack of resistance to PIs in this study may be due to the high genetic barrier of PIs. A previous TDR survey in northern Taiwan showed that 2.3% of treatment-naïve HIV-infected patients demonstrated resistance to PIs [12]. A low prevalence of TDR to PIs has also been reported in Europe and the USA, with rates ranging from < 1 to 2.7% [27–29]. As PIs are also suggested as a first-line therapy by some guidelines, active monitoring of PI resistance may still be necessary.

In the present study, the most common drug resistance mutation observed in the patients was K103 N, which was found in 6 individuals. A total of 4 K103 N variants were detected after 2012 when the fixed regimen policy was introduced. K103 N causes high-level resistance to NVP and EFV. Although there was no significant change in the prevalence of K103 N mutations before and after 2012 (*P* = 0.32), the K103 N TDR should be monitored closely because NNRTIs are still used as a first-line ART in Taiwan. The slight increase in the prevalence of K103 N TDR may be a consequence of the introduction of a fixed regimen with NNRTIs as the first-line therapy in Taiwan during the study period. Several previous studies have reported an association between the widespread use of NNRTIs and an increase in K103 N TDR [30, 31]. In addition, virology studies have shown that K103 N, a major NNRTI mutation, can persist for a long time in the absence of treatment [32]. This is because K103 N only has a limited effect on replicative capacity [33]. As NNRTIs are still widely prescribed as a once-daily single tablet regimen, and combination ART consisting of 2 NRTIs and 1 NNRTI remains the recommended first-line regimen in the World Health Organization treatment guidelines for adults [34], close monitoring of the prevalence of K103 N mutations is important for further evaluation of first-line ART options and their effects on TDR.

Both V179D and K103R are polymorphisms that by themselves do not predict treatment failure of EFV-based regimen. However, the combination of V179D and K103R can have a synergistic effect to reduce susceptibility to NVP and EFV [17]. In the present study, the prevalence rates of V179D and K103R were 5.3 and 37.1%, respectively. There was no significant increase in the prevalence of V179D or K103R between the two study periods (results not shown). Five individuals were found to have the combination V179D and K103R, and four-fifths of them were detected after 2012. There was a high prevalence rate of K103R in our patients, however Harrigan et al. [35] reported that K103R substitutions most likely represent naturally occurring polymorphisms in HIV reverse transcriptase, and that they are not directly associated with NNRTI exposure or resistance. However, the presence of two or more polymorphisms may be associated with a higher risk of virologic failure [36]. The clinical impact of these polymorphisms is unclear, and larger datasets may help to elucidate this issue.

The median viral load was higher during period 2, which may be because the VCT attendees were more aware of their physical condition and were seeking an evaluation during the primary HIV infection. As the patients were diagnosed following VCT, it was not possible to trace back their previous test results. However, highly overlapping confidence intervals means that the results may not be clinically significant. An extremely high viral load (> 1,000,000/copies) was detected more often during period 2 (5/161) compared with period 1 (11/216). No significant differences were observed between age, CD4 count, gender and HBV, HCV coinfection in the patients from period 1 and 2.

There are some limitations to the present study. First, the follow-up period was relatively short and may mask the real effect of the fixed ART regimen. We hypothesized that a fixed regimen with AZT/3TC plus EFV or NVP would increase the prevalence of TDR in Taiwan. However, the fixed regimen policy only lasted for 4 years, and the TDR remained stable during this time period. Therefore, the true effect of a fixed regimen with AZT/3TC plus EFV or NVP should be interpreted carefully. Second, the study population were VCT clients, and most were MSM. Although MSM are the group reported to be at most risk of HIV infection in Taiwan (around 50~60%), the present study may still not reflect the whole picture of TDR in southern Taiwan. Third, we did not perform specific tests to prove recent HIV infection in our population. However, all of our participants were enrolled from VCT services, and were therefore more likely to be in an early stage of HIV infection. In addition, pre-exposure prophylaxis is available in Taiwan after 2016. Therefore, the drug resistance was less likely to be acquired due to prior ART drug exposure. Finally, this is a single center study, and the conclusions need to be verified in large multicenter studied in Taiwan.

## Conclusions

In conclusion, a fixed regimen with AZT/3TC plus EFV or NVP as first-line therapy for HIV-infected treatment-naïve patients did not increase the TDR in southern Taiwan during a 4 year follow-up period.

## Data Availability

The datasets used and analysed in the current study are available from the corresponding author on reasonable request.

## Abbreviations

3TC: Lamivudine
ART: Antiretroviral therapy
AZT: Zidovudine
EFV: Efavirenz
FTC: Emtricitabine
HBV: Hepatitis B virus
HCV: Hepatitis C virus
HIV: Human immunodeficiency virus
INSTI: Integrase strand transfer inhibitor
MSM: Men who have sex with men
NNRTI: Non-nucleoside reverse transcriptase inhibitor
NRTI: Nucleoside reverse transcriptase inhibitor
NVP: Nevirapine
PI: Protease inhibitor
RT-PCR: Reverse transcription-polymerase chain reaction
TDR: Transmitted drug resistance
VCT: Voluntary counseling and testing
